# Computerized tomography angiography in preoperative assessment of intravenous leiomyomatosis extending to inferior vena cava and heart

**DOI:** 10.1186/s12885-016-2112-9

**Published:** 2016-02-08

**Authors:** Ting Gui, Qiuhong Qian, Dongyan Cao, Jiaxin Yang, Ping Peng, Keng Shen

**Affiliations:** Department of Obstetrics and Gynecology, Peking Union Medical College Hospital, Peking Union Medical College, Chinese Academy of Medical Sciences, Beijing, 100730 China

**Keywords:** Computerized tomography angiography, Intravenous leiomyomatosis, Inferior vena cava, Heart, Surgery

## Abstract

**Background:**

Intravenous leiomyomatosis (IVL) extending to inferior vena cava and heart is one of the most challenging conditions for surgical treatment. We explored the use of computerized tomography angiography (CTA) in preoperative assessment for this disease.

**Methods:**

A cohort of 31 patients with IVL extending to inferior vena cava and heart were reviewed from the year 2002 to 2014, focusing on the preoperative CTA imaging characteristics and the surgical procedures in clinical treatment.

**Results:**

All patients were diagnosed correctly combining the clinical medical history and CTA imaging. Thirteen patients had tumors confined within the inferior vena cava, and 18 patients had tumors intruding into the right heart. Furthermore, 15 tumors were located in the right atrium alone, and 3 tumors involved both the right atrium and the right ventricle. All patients had simple or multiple soft tissue masses from the pelvis, with 22 tumors extending into inferior vena cava through the iliac veins and 9 tumors through the ovarian veins. Three patients had tumors invading into lung and underwent tumor thrombus resection in the pulmonary artery. Patients received either one-stage surgery or two-stage surgery dependent on patient general condition and tumor status. All operations were successfully performed by multidisciplinary cooperation, including gynecology, cardiac surgery, and vascular surgery, without severe surgical-related complications or deaths.

**Conclusions:**

CTA imaging can present location, size, and full-scale extension pathway of IVL lesions, and can be used as first-line imaging technique in preoperative assessment, having great significance in making surgical plan and obtaining successful outcome.

## Background

Intravenous leiomyomatosis (IVL) is an extremely rare form of leiomyoma [1]. IVL, originating from either a uterine leiomyoma or the smooth muscle layers of uterine veins, is recognized as benign histologically but aggressive in behavior characterized by intravascular tumor invasion [2]. Because of its rarity, and the fact that patients are usually asymptomatic until the tumor extends into the heart, early diagnosis is difficult and uncommon [3].

The tumor primarily extends through the uterine veins, sometimes reaching the inferior vena cava, the right cardiac chambers, and even the pulmonary artery [4]. Cardiac-extending IVL is one of the most challenging conditions for surgical treatment because complete tumor resection is the key to manage the disease. It often requires significantly invasive surgery such as cardiopulmonary bypass or two-step surgery [5]. Unplanned reoperation, disseminated intravascular coagulopathy, massive transfusion due to large blood loss, and patient mortality have been reported as the surgical complications [6].

Therefore, early diagnosis and comprehensive preoperative assessment is crucial in clinical management of IVL extending to inferior vena cava and heart. The purpose of our study is to assess the role of computed tomography angiography (CTA) in preoperative evaluation including delineating tumor extent and making surgical plan for this disease.

## Methods

From the year January 2002 to December 2014, 51 patients with IVL were admitted to PUMCH, 31 (60.8 %) patients diagnosed as IVL extending to inferior vena cava and heart. Patients complaining of cardiac symptoms in addition to abdominal/pelvic mass, such as chest tightness, shortness of breath, palpitation, swelling of lower extremities, and even syncope, subsequently underwent CTA if ultrasound or echocardiography indicated space-occupying lesions inside inferior vena cava and heart.

CTA was conducted by using a 64-slice multidetector computerized tomography (Siemens Somatom Sensation 64 slice, Siemens Medical Solutions, Forchheim, Germany). Image acquisition on the 64-slice scanner was performed with the following parameters: tube voltage 120 kV; effective mAs 450; slice collimation 0.6 mm; pitch 0.625; rotation interval 0.25–0.30 mm; the field of view from pelvic floor to 3 cm above tracheal carnia. Two experienced radiologists retrospectively assessed data sets using a workstation (Philips EBW), which allowed interactive postprocess of imaging data including volume rendering (VR), volume intensity projection (VIP), maximum intensity projection (MIP), multi-planar reconstruction (MPR), and curve-planar reconstruction (CPR). All reconstructed image were excellent.

Information on the location, size, morphology, density, blood supply, contrast, extension pathway, and adhesion of the tumor would be offered. After comprehensive preoperative evaluation, patients with IVL extending to inferior vena cava and heart were submitted to one-stage surgery or two-stage surgery. Surgeries were performed by experienced surgeons comprising gynecologists, cardiac surgeons, vascular surgeons, and urinary surgeons. Final diagnoses were confirmed by at least two pathologists based on the postoperative histopathological findings, in combination of imaging demonstration and surgical findings.

### Ethics statement and consent statement

Our research was approved by the Ethics Committee of Peking Union Medical College Hospital (PUMCH). Written informed consent to use individual clinical data for publication was obtained from each patient.

## Results

The median age of the entire study population was 31 years (range 24–51 years). The median gravida and para of the patients was 3 (range 0–6) and 1 (range 0–4), respectively. All the patients had history of uterine leiomyoma, and 21/31 (67.7 %) had undergone an operation of uterine leiomyoma, either myomectomy or hysterectomy.

Patients who were suspicious of uterine leiomyoma and complained of cardiac symptoms such as chest tightness, shortness of breath, edema of lower extremities were submitted to CTA. Combining the features of CTA imaging and history of uterine leiomyoma, the diagnosis of IVL was considered before surgery. The preoperative diagnostic coincidence rate was 100 % compared with postoperative pathological review.

From CTA images, first we could observe simple or multiple space-occupying lesions in the pelvis. The primary tumor lesions of IVL are specifically manifested by augmentation and tortuous clustering of uterine or parametrial vessels. The surface of tumors intruding into venous lumen are covered by smooth inima or endothelium, inadhesive to the venous walls, thus characteristically presented as free mass within the vascular system. In the coronal plane, tumor lesions in the pelvis and in the inferior vena cava are continuous and inhomogeneously enhanced, shaped like “sausages”, and when the right heart are involved, we could observe characteristic “walking stick head” or “snake head” tumor masses. In the cross-sectional plane, the lumen of inferior vena cava presented circular or semicircular change, sometimes with calcification of tumor lesions.

**Fig 1:**
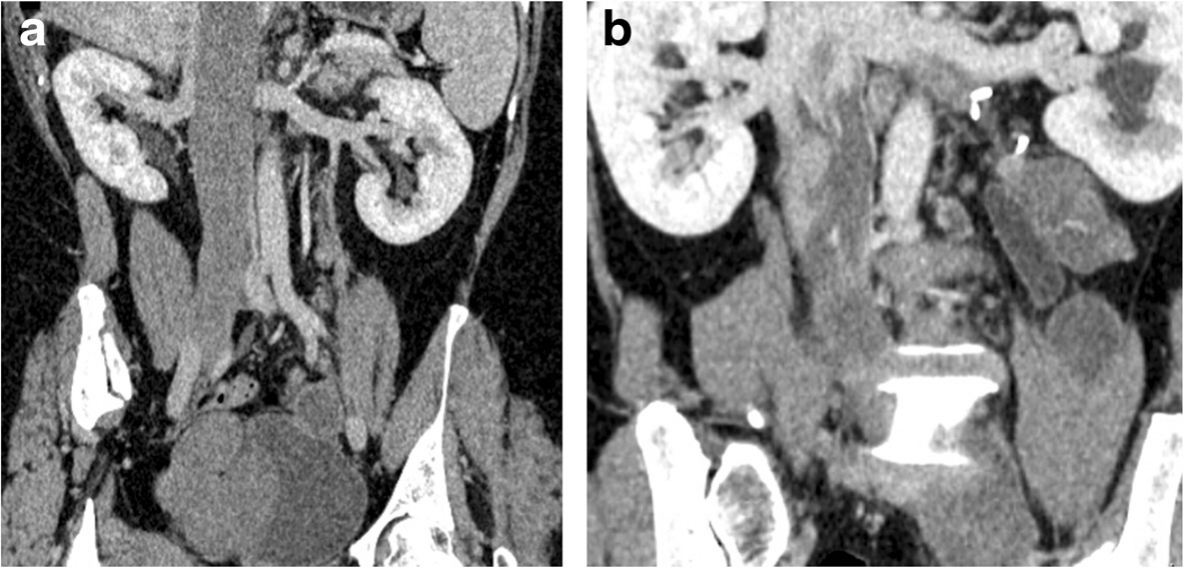
Two extension pathways of intravenous leiomyomatosis. **a** Coronal image showed filling defect in the enlarged inferior vena cava extending from the right common iliac vein. **b** Coronal image presented tumor extension pathway through left ovarian vein

**Fig 2:**
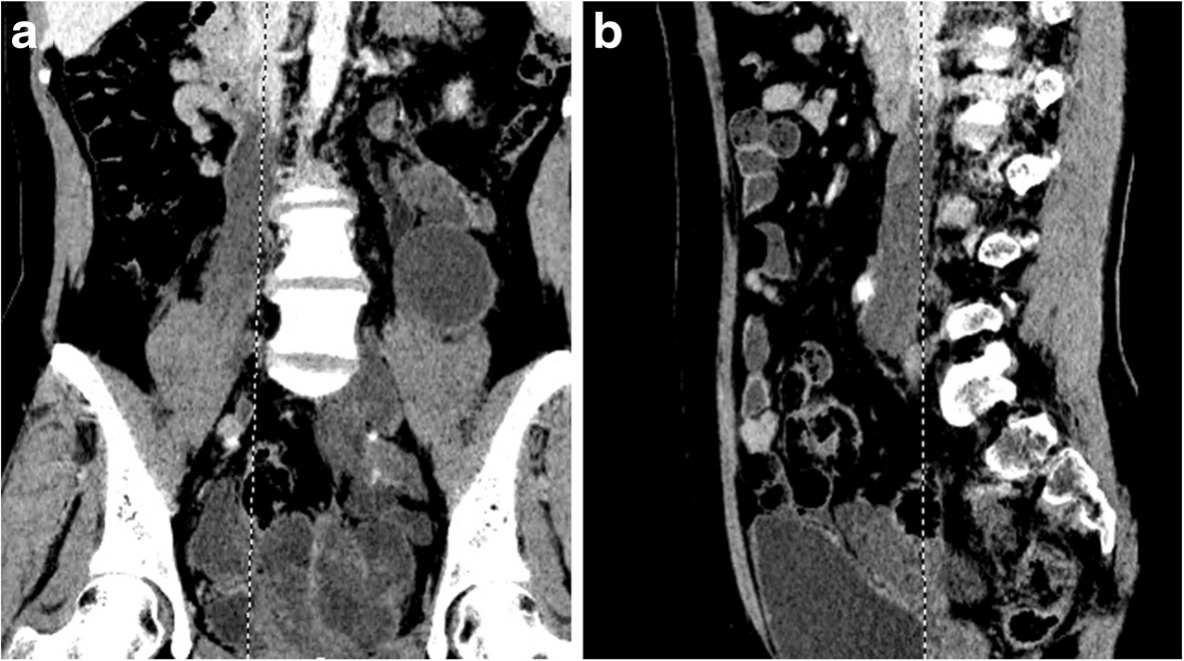
Intravenous leiomyomasis in the pelvis extending into the inferior vena cava. **a** Coronal image showed large tumor mass in the pelvis with heterogeneous enhancement and filling defect in the enlarged inferior vena cava with free anterior extremity. **b** Sagittal image presented spindle-shaped filling defect confined within the inferior vena cava

**Fig 3:**
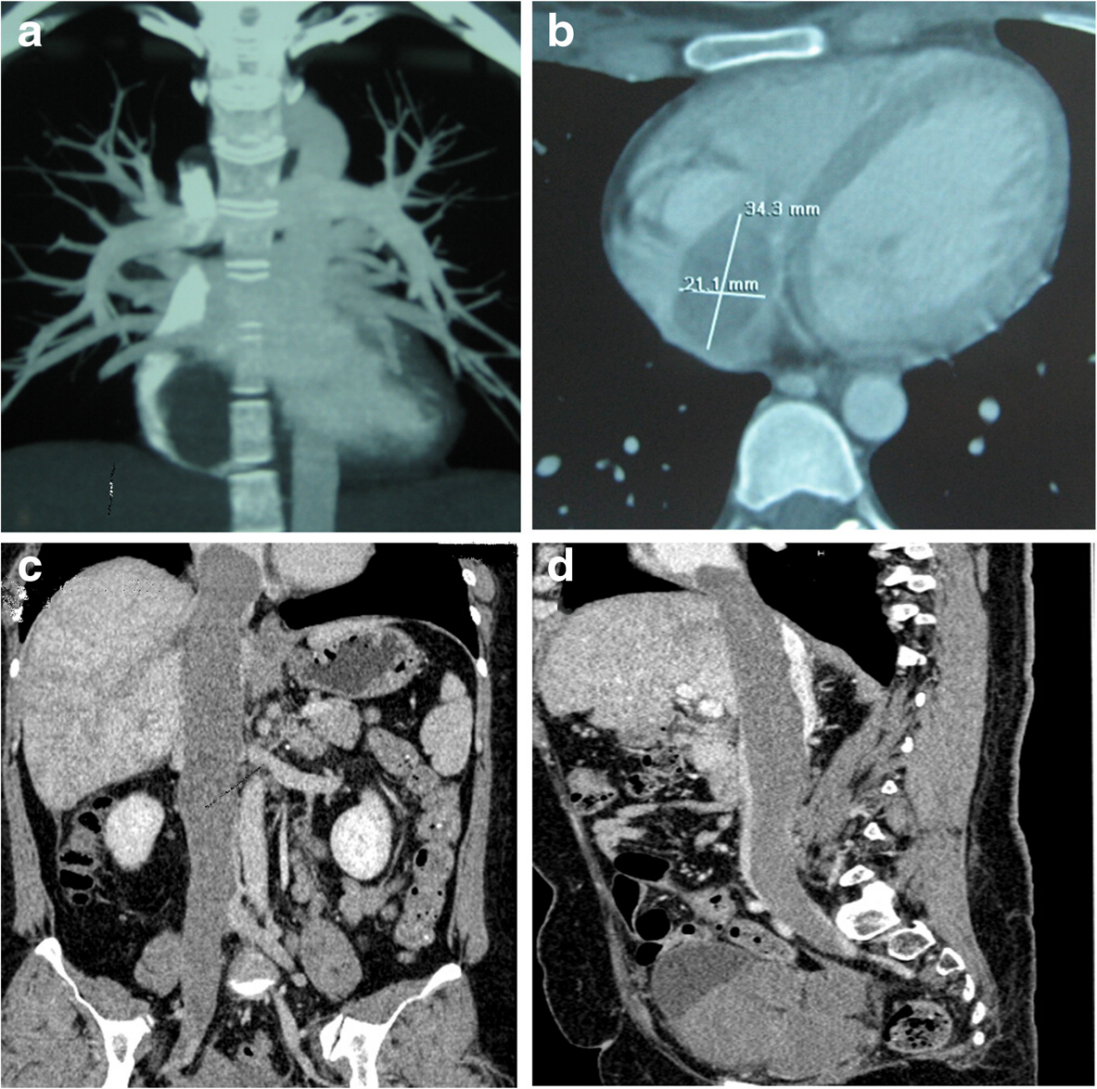
Intravenous leiomyomatosis extending into the right heart. **a** Coronal image demonstrated large filling defect in the right heart. **b** Transverse image showed the tumor mass in the right atrium as an elliptical shape filling defect. **c** Coronal image presented large filling defect in the inferior vena cava extending from the right common iliac vein and to the right heart like a walking stick head. **d** Sagittal image demonstrated tumor mass in the pelvis and large filling defect in the enlarged inferior vena cava and the right heart like a snake head

A distinct advantage of CTA is directly presenting the full-scale path of extension. Patients with IVL usually had simple or multiple soft tissue masses from the pelvis extending into the inferior vena cava. In our study, 22 patients had tumors extending into inferior vena cava through the iliac veins, and 9 patients through the ovarian veins (Fig. 1). The masses in the pelvis and in the veins were continuous. The anterior extremity was free, and the margin was regular with clear boundary. Of the 31 patients, 13 patients had tumors confined within the inferior vena cava (Fig. 2), and the other 18 patients had tumors intruding into the right heart (Fig. 3). Furthermore, 15 (83.3 %) tumors were located in the right atrium alone, and 3 (16.7 %) tumors involved both the right atrium and the right ventricle. When the right heart was involved, the lesions presented with characteristic “walking stick head” or “snake head” shape (Fig. 3).

Additionally, among our study population, three patients had tumors invading into lung and underwent tumor thrombus resection in the pulmonary artery. Besides, six patients had renal involvement and defect of renal function, because the pelvic mass compressed the ureter and caused hydronephrosis.

It is noteworthy that, in our study, of the 31 patients, 26 patients underwent ultrasonography in multiple regions before submitted to CTA, and the other 5 patients received MRI before performing CTA.

The technical requirement for ultrasonography was relatively lower, but the information given by ultrasonography was fragmented compared with CTA. Pelvic ultrasound helped us finding single or multiple solid tumors in the pelvic or even abdominal cavity; from abdominal vascular ultrasound, we could observe “vessel-in-vessel” sign in the inverior vena cava, iliac veins, and renal veins; and ultrasonic cardiogram was able to tell us if there were space-occupying lesions in the right heart, or even the pulmonary artery, in addition to the morphology and structure of the heart. Comprehensive analysis of these fragmented information could help us making diagnosis. However, the continuity and extension pathway of tumors was not available, which was cruicial for making surgical plans.

Regarding MRI, its scanning range could be as large as that of CTA, and the post-processing techniques also could provide three-dimensional images. However, the spatial resolution of image was relatively poor containing more artifacts, and the details of intravascular diseases cound not .be presented as clear as compared with CTA. Besides, the time needed for examination was much longer, and patients carrying cardiac pacemaker or metal intrauterine were prohibited from this examination.

With the information offered by CTA, surgical plans were subsequently made after multidisciplinary consultation dependent on the tumor extent and patient general condition.

**Fig 4:**
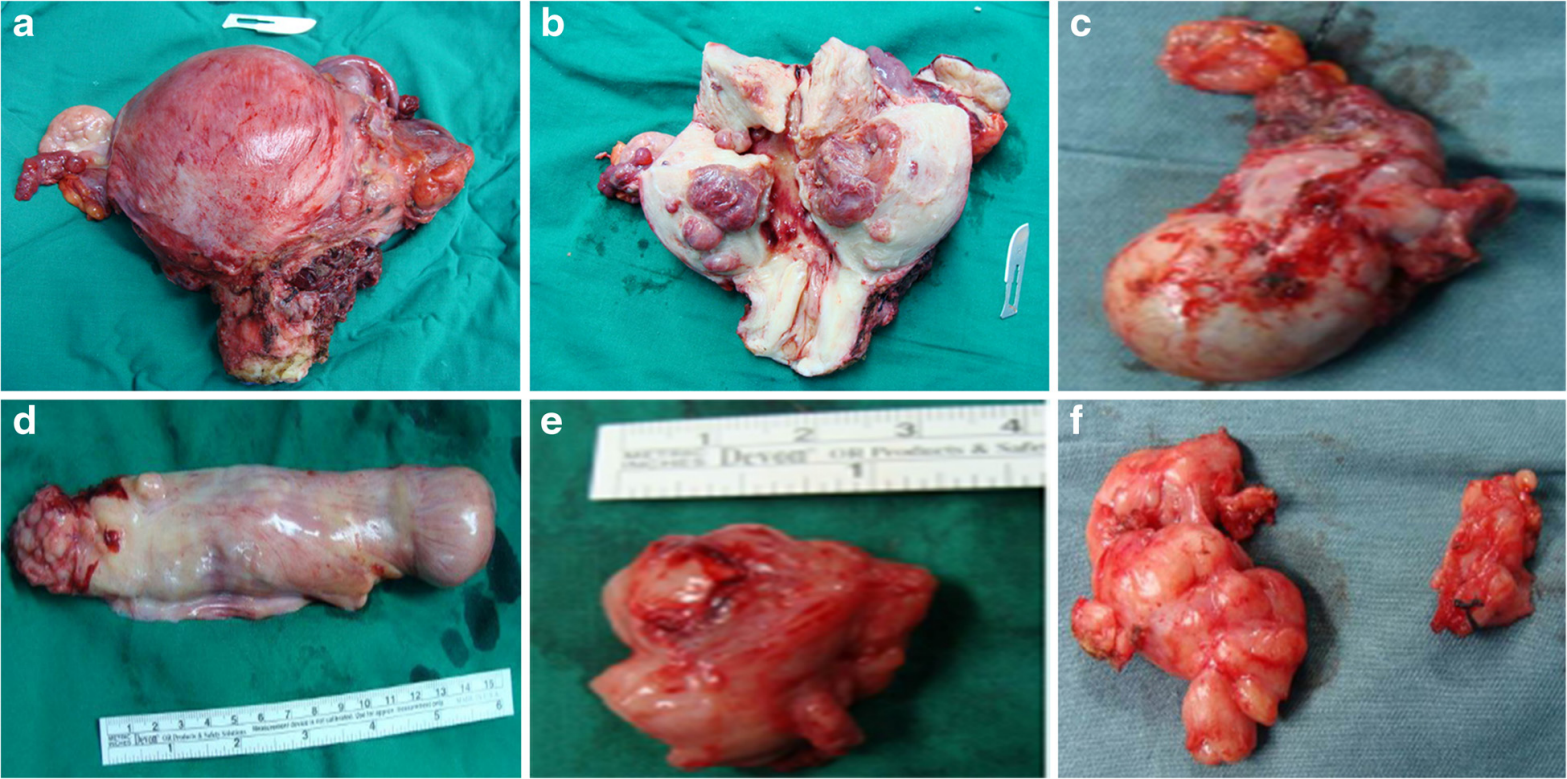
Surgical specimens removed from patients with intravenous leiomyomatosis extending through iliac vein to inferior vena cava and heart. **a** Anterior view of uterus. **b** Posterior view of uterus. **c** Left adnexa. **d** Tumor in the inferior vena cava and the right heart. **e** Tumor in the common iliac vein. **f** Pelvic retroperitoneal mass

Fifteen patients underwent one-stage operations including tumor thrombus resection from the inferior vena cava, pelvic and abdominal mass resection, total abdominal hysterectomy and bilateral salpingo-oophorectomy with or without intracardiac tumor resection (Fig. 4). Of the 15 patients, 7 patients were submitted to sternotomy and pericardiotomy for resection of large tumors in the heart, and 8 patients received venotomy only without open heart surgery for tumor thrombus resection from the inferior vena cava. The other 16 patients were submitted to two-stage operations, that was cardiac surgery first and then vascular and gynecologic surgery. The procedures were divided by the level of renal vein. In the first-stage operation, 10 patients with cardiac extension underwent sternotomy and pericardiotomy, and 6 patients without cardiac extension just received tumor thrombus resection from the inferior vena cava above the renal vein level through venotomy. In the second-stage operation, the procedures including tumor thrombus resection from the inferior vena cava below the renal vein by venotomy, pelvic and abdominal mass resection, and total abdominal hysterectomy with bilateral salpingo-oophorectomy were performed simultaneously.

All operations were successfully performed by multidisciplinary cooperation, including department of gynecology, cardiac surgery, and vascular surgery, without severe surgical-related complications or deaths.

## Discussion

Intravenous leiomyomatosis is a special type of uterine myoma which grows in the venous system. The first case of IVL extending to inferior vena cava and heart was reported by Durck in 1907 [7]. Preoperative diagnosis is often difficult because the clinical manifestations are various and nonspecific [8]. If patients have a uterine tumor complicated by chest tightness, shortness of breathing, swelling in the lower extremity, and even paroxysmal syncope, the diagnosis of IVL extending to inferior vena cava and heart should be suspected [9].

Concerning the origination of IVL, there have been two theories: the first is from smooth muscles cells in the venous walls, which proliferate and intrude into venous lumen; the second is from uterine myoma, benign tumor cells invading uterine veins and continuing to grow in the inferior vena cava [10, 11]. Most reports in the literature support the uterine myometrium origination theory for the following reasons. First, patients with IVL had history of uterine myoma or history of myomectomy or hysterectomy. Second, gross histological examination and imaging examination found that tumors intruded into uterine veins shaped like a worm or a finger not adhesive to the venous walls, and the bases of tumors were connected with the wall of uterus. Third, immunohistochemical examination indicated that tumor cells were estrogen- and progesterone-receptor positive, while the smooth muscle cells in the venous walls were estrogen- and progesterone-receptor negative.

When IVL extends into inferior vena cava and heart, or even pulmonary artery, the life of patients will be threatened fatally, that is the malignant behavior of IVL. Tumor growth in the heart can increase the risk of sudden death attributable to the tricuspid valve orifice or right ventricular outflow tract obstruction, thus early diagnosis and timely operation are of great importance in improving patient outcome [12]. Comprehensive preoperative evaluation is crucial for making surgical plans. Preoperative imaging that can offer precise information about the full-scale of tumor and its relationship with adjacent tissues is of great necessity.

Optimal diagnostic management of IVL remains a topic of ongoing debate. Typical imaging investigation for IVL encompasses different methods including ultrasonography, magnetic resonance imaging (MRI), and computed tomography (CT). Ultrasonography, especially cardiac ultrasound, is useful in evaluating intravascular and intracardiac lesions when conducted by an experienced operator, but requires highly specialized training. Three-dimensional MRI is playing an increasingly important role in modern imaging diagnosis, but it presented with relatively poor spatial resolution and more artifacts, and is time-consuming and not suitable for patients carrying cardiac pacemaker or metal intrauterine device. The availability of a non-invasive readily available diagnostic technique is therefore desirable.

Recent advances in CT have enabled the acquisition of three-dimensional data sets with submillimeter spatial resolution, allowing for detailed image reconstruction which resembles conventional angiography. Besides, multi-slice computed tomography enabled the acquisition of CT angiographic data sets over an extended field of view including abdomen, pelvis, and lower extremities in only a few seconds with a single contrast bolus [13]. In addition, post-processing techniques could present an overall and visual view of the tumor size, location, and extension pathway [14].

Compared with ultrasonography and MRI, a distinct advantage of CTA is directly presenting the full-scale path of tumor extension. The chest & abdomen & pelvis combining scans could clearly display IVL extension pathway. There are two classical pathways: the first is from uterine vein, iliac vein, to inferior vena cava [15–17]; the second is from ovarian vein, left renal vein, to inferior vena cava [18]. In addition, the condition of other organs could also be revealed by CTA, such as hydronephrosis, pulmonary or hepatic metastasis, abdominal ascites, or pericardial effusion, as well as collateral circulation.

In the literature, most IVL tumors grow unilaterally, occasionally growing bilaterally. Over 50 % of IVL tumors extend through internal iliac vein, about 25 % extend through ovarian vein, and about 5 % extend through both internal iliac vein and ovarian vein [3]. In our study, 70.9 % patients had IVL extending into inferior vena cava through iliac veins, and for the other 29.1 % patients, ovarian vein provided an alternative route to the subphrenic segment of the inferior vena cava.

Surgery is the only effective treatment modality for IVL extending to inferior vena cava and heart. Resection includes the uterine tumors, parametrium tumors, ovaries and fallopian tubes, and intravenous and intracardiac tumors. Various surgical approaches have been used for removing the IVL tumor, mainly including one-stage surgery and two-stage surgery. In 1982, Ariza et al reported intracardiac components resection followed by a delayed laparotomy [19]. Since then, most researchers have adopted the two-stage surgical procedure, which separates abdominal-pelvic surgery and chest surgery. But a series of successful one-stage surgeries have also been reported [20, 21]. In our study, we prefer the two-stage surgery be performed for patients with poor general condition who cannot tolerate a whole multidisciplinary operation simultaneously; one-stage surgery could be adopted for patients with good performance status and small tumors in the cardiac chamber. If the tumor has a smaller diameter than the inferior vena cava, grows freely without adhesion to the vascular walls, and is only located in the right atrium without extension to the right ventricle, venotomy could be adopted to remove the tumor directly through the inferior vena cava under intraoperative transesophageal echocardiography. If the tumor in the cardiac chamber is too large or extends into the right ventricle, sternotomy and pericardiotomy will be needed. Two-stage surgery is divided into operations above and below the renal vein. In the first-stage surgery, sternotomy, pericardiotomy, and venotomy will be used to remove the tumor in the right heart and the upper inferior vena cava. In the second-stage surgery, venotomy is adopted to remove tumor thrombus in the inferior vena cava below the renal vein, and gynecological procedures are performed together. Multidisciplinary cooperation among cardiac surgery, vascular surgery, and gynecologic surgery helps guarantee a successful outcome.

The development of cardiopulmonary bypass revolutionized cardiac surgery and contributed immensely to the improvement of patients outcomes [22]. In our study, to remove IVL tumors in the right ventricle, three patients received cardiopulmonary bypass, allowing direct visualization of the inside of the opened human heart. Cardiopulmonary bypass exposes patients to a complex set of nonphysiological functional alterations [22]. Haemolysis, ischaemia, and perfusion injury and neutrophils activation during cardiopulmonary bypass play an important role in oxidative stress of multiple organs such as the myocardium, lungs, and kidneys [23]. The administration of agents with antioxidant properties during surgery may reduce reactive oxygen species burst and oxidative stress [23]. Although the cardiopulmonary bypass is not perfect, it is still an essential part of cardiac surgery.

Differential diagnosis should be performed between IVL and other diseases that have similar CT imaging that is space-occupying lesions in the venous system or right cardiac and pulmonary system. The differential diagnosis of IVL mainly includes intravenous thrombus, Budd-Chiari syndrome, right atrial myxoma, primary leiomyosarcoma, endometrial stromal sarcoma [24]. First these diseases usually do not have the history of uterine myoma. Intravenous thrombus has no enhancement in contrast CT because no vessels growing inside. In patients with Budd-Chiari syndrome, we could observe occupying lesions in the hepatic vein and hepatic segment of inferior vena cava, causing partial or compete vascular obstruction. Patients mainly present with liver and spleen enlargement, severe ascites, varicosity in the thoracic and abdominal walls as well as lower extremities, while patients with IVL do not complain of varicosity. Right atrium myxoma locates only in the heart chamber, some intruding into pulmonary artery, and generally does not affect the IVC. Primary leiomyosarcoma was first reported in 1896, with a ratio of male over female 1:5. Most tumors are malignant and originate from inferior vena cava. The differential diagnosis between primary leiomyosarcoma and IVL is very difficult at early stage, because symptoms are nonspecific and various. When patients have obvious symptoms, metastases to liver, lung and retroperitoneal lymph nodes often have occurred. From contrast CT, we could observe inhomogeneous enhancement of tumor lesions, invasion of vascular walls and adjacent tissues, and abundant collateral circulation, while IVL tumors usually have clear boundary and are not adhesive to vascular walls. Endometrial stromal sarcoma is a kind of tumor with low malignancy, and could be observed at any age stage with most patients not yet reaching menopause age. It could invade small veins and lymphatic vessels, and has overlap in histological and immunohistochemical examination compared with IVL. However, endometrial stromal sarcoma tissues have plenty of spiral arteries and are positive in CD10. Moreover, whereas IVL occurs only in women, leomyosarcoma can also be found in males [25–27]. Although these features help the diagnosis of IVL, the final diagnosis depends on histopathology.

## Conclusions

In conclusion, CTA can present location, size, and full-scale extension pathway of IVL lesions, and can be used as first-line imaging technique in preoperative assessment, having great significance in making surgical plans and obtaining successful outcome.

### Availability of data and materials

All the data supporting our findings could be found in this paper.

## Abbreviations

CPR: curve-planar reconstruction
CT: computerized tomography
CTA: computerized tomography angiography
IVL: intravenous leiomyomatosis
MIP: maximum intensity projection
MPR: multi-planar reconstruction
MRI: magnetic resonance imaging
PUMCH: Peking Union Medical College Hospital
VIP: volume intensity projection
VR: volume rendering
